# HPV16 persistent infection and recurrent disease after LEEP

**DOI:** 10.1186/s12985-019-1252-3

**Published:** 2019-11-27

**Authors:** Maria Teresa Bruno, Nazzario Cassaro, Salvatore Garofalo, Sara Boemi

**Affiliations:** 1 Department of General Surgery and Medical Surgery Specialities, Gynecological Clinic of the University of Catania, Policlinico. Via S. Sofia78, Catania, Italy; 2 Gynecological Oncology, Humanitas, Catania, Italy

**Keywords:** Papillomavirus infection, LEEP, CIN2+, Relapse, Recurrent desease, Positive margin

## Abstract

**Background:**

About 23% of patients develop CIN2+ after LEEP treatment due to residual or recurrent lesions. The majority of patients with HPV infection were HPV negative before treatment, but 16,4% were still HPV 16 positive after treatment, indicating that conization do not necessarily clear HPV infection rapidly. The aim of this retrospective study was to evaluate the possible correlation existing between the appearance of recurring high-grade lesions and the viral genotype 16, and other risk factors such as residual disease.

**Methods:**

One hundred eighty-two HPV positive patients underwent LEEP for CIN2+. The follow-up post treatment was carried out every 6 months. Abnormal results during follow-up were confirmed histologically and considered recurrent high-grade intraepithelial cervical lesions (CIN2/CIN3 or CIS).

Statistical analysis was performed by using the SPSS software package for Windows (version 15.0, SPSS, Chicago, IL, USA). Descriptive statistics are expressed as frequency, arithmetic mean, standard deviation (S.D.) and percentages. We calculated significance (*P* < 0.5) with the Easy Fischer Test. We calculated the Odds Ratio (OR) of women with peristent HPV 16 infection and positive margin, to have a recurrence.

**Results:**

In our study, the rate of persistent infection from HPV 16, after LEEP, was 15.9% (29/182) with 94% (17/18) of the recurring disease occurring within 18 months of follow up. From this study it was found that the persistence of genotype 16 is associated with a greater rate of relapse post-conization of CIN 2+ lesions, with respect to other genotypes. Our study further supports those studies that demonstrate that the risk for residual disease or relapse is not to be overlooked, also when the margins are negative, but persistent HPV infection is present. In our case study, 40% of relapses were in women with negative margin, but with persistent HPV 16 infection. Even more so, the margins involved in HPV16 positive subjects is another prediction factor for relapse.

**Conclusions:**

Our results show the importance of genotyping and that persistent HPV 16 infection should be considered a risk factor for the development of residual/recurrent CIN 2/3.

## Background

The treatment of Cervical Intraepithelial Neoplasia grade 2+ (CIN2+) (CIN3-CIS) consists in a conservative surgical approach using loop electrosurgical excision procedure (LEEP) that guarantees surgical radicality and preserves functional integrity of the uterine cervix, considering that young women are the most affected by these pathologies. The treatment for CIN 2/3 or carcinoma in situ (CIS) with LEEP is efficacious and most patients require no further treatment. However, about 23% of patients develop CIN2+ after conservative treatment due to residual or recurrent lesions [1].

It has been seen that the women who do not eliminate the virus have greater recurrence rates. Furthermore, in the ASC-US LSIL triage study (ALTS trial) [2] and in the more recent study of Heymans et al. [3], patients with HPV 16 were identified as being at high risk of recurrence and thus were given a more frequent follow-up.

Based on these data, in this study we wanted to investigate if the HPV 16 genotype could be a risk factor with respect to high-grade lesion recurrence after excisional treatment with LEEP and the management of the residual disease in cases of cone with positive margin.

## Aim of the study

The aim of this retrospective study was to evaluate the possible correlation existing between the appearance of recurring high-grade lesions and the viral genotype 16, and other risk factors such as residual disease.

## Materials and methods

The study protocol was approved by the Institutional Review Board of the Department and was conducted. in accordance with the 1975 Declaration of Helsinki.

We studied the clinical files of 230 patients who, from April 2015 to April 2017, underwent LEEP for CIN2+ at the Colposcopy Outpatient Service of the Gynaecological/Obstetrics Unit at the Policlinico Universitario, Catania (University of Catania, Italy) and who satisfied the following inclusion criteria:

- Patients with histological diagnosis of CIN2+;
- Patients who had an HPV test before and after treatment;
- Patients who had no anti-HPV vaccination;
- Patients without pathologies of the immune system;
- Patients who had completed at least two-years of follow-up.

Moreover, patients were excluded if they were HPV negative or had any suspicion of infiltrating neoplastic pathologie.

Only 192 patients satisfied the inclusion criteria; their clinical data was collected, of which: patient’s age, type of pathology, HPV strain, treatment method used, resection margins, follow-up cervical histology, date of follow-up, pretreatment viral genotype, post-treatment HPV genotype, and recurrent cases.

All the patients underwent conservative surgical treatment of cone excision (conization) electrosurgery with conization (LEEP) of 20 mm width and 12, 15 or 20 mm depth (Utah Medical Products, Midvale, Utah, USA).

The histological examination of the cervical cone established a definitive histological diagnosis and evaluated the extension of the cone margins, defined as positive if the distance between the CIN lesion and the margin of the resection was less than 1 mm.

The follow-up was carried out every 6 months during the first year after treatment and then once to year (At the moment of excisional treatment (T0) and the successive follow-ups at 6 (T1), 12 (T2), 18 (T3) and 24 (T4) and 30 (T5) months). Abnormal results during follow-up were confirmed histologically and considered recurrent high-grade intraepithelial cervical lesions (CIN2/CIN3 or CIS).

At each follow-up examination the patients were examined by conventional Pap test, HPV test and colposcopy, moreover, cytological eso-endocervical samples were taken and placed in ThinPrep Solution. The samples were sent to the laboratory for extraction of total DNA for the genotyping of viral DNA by means of genetic amplification followed by hybridization with genotype-specific probes able to identify most HPV genotypes of the genital region [28 genotypes of high-risk HPV (16, 18, 26, 31, 33, 35, 39, 45, 51, 52, 53, 56, 58, 59, 66, 68, 73, 82), low-risk (6, 11, 40, 43, 44, 54, 70) and not defined risk (69,71,74)].

The commercial method used was NucliSenseasy MAG system bio Merieux SA, Marct l’Etoile, France.

The HPV genotype was classified as follows: 1) negative, 2) HPV 16, 3) hr HPV (HPV18, 31, 33, 45 and other hr HPV).

Colposcopy was carried out using a Zeiss OPM1F colposcope (Carl Zeiss, Jena, Germany) and applying acetic acid and a Lugol iodine solution. Any colposcopic abnormality was classified based on the nomenclature proposed by the International Federation for Colposcopy and Cervical Pathologies (IFCPC) in 3 grades of increasing anomalies based on severity: Zone of Abnormal Transformation (AnTZ) grade 1 (AnTZ1) and grade 2 (AnTZ2), or cancer. We evaluated the visibility of the squamo-columnar junction and specific biopsies were taken from the portio and/or endocervical curettage to guide the diagnosis in cases of an abnormal Pap test or suspicion of residual/recurrent disease at colposcopy.

### Statistical analysis

Statistical analysis was performed by using the SPSS software package for Windows (version 15.0, SPSS, Chicago, IL, USA). Descriptive statistics are expressed as frequency, arithmetic mean, standard deviation (S.D.) and percentages.

We calculated significance (*P* < 0.5) with the Easy Fischer Test.

We calculated the Odds Ratio (OR) of women with peristent HPV 16 infection and positive margin, to have a recurrence.

## Results

The mean age of the 192 patients was 39.3 ± 8.7 years (range 22 to 73 years). During the follow-up, 18 (9.8%) of the 182 patients relapsed. The average time between LEEP and the diagnosis of recurrent disease was 18 months (range 6 to 30 months).

The 192 patients had CIN3-CIS, as determined at biopsy or histological examination of the cone. In total, 182 (94.7%) of the 192 patients were positive for the HPV test before surgery, 10 patients that were negative at the HPV test excluded by the study.

Of the 182 patients with a positive HPV test, 104 (57.1%) belonged to genotype 16, 78 (42.8%) to other genotypes. (Table 1).

**Table 1:** patients before treatment

HPV Test	Preoperative patients	
HPV 16	104	57.1%
hrHPV	78	42.8%

Of the 182 patients positive at the HPV test at T0 (before LEEP), 144 (79.1%) were negative at 6 months (T1) (*p* < 0.001), 29 cases (15.9%) HPV 16 showed virus persistence, only 9 case of virus persistence was found in the hr HPV group (Table 2).

**Table 2:** Patients after treatment

HPV Test	Postoperative patients	
HPV 16	29	15.9%
hrHPV	9	4.9%
Negative HPV	144	79.1%

A negative margin was found in 162/182 (89%) patients, while 20/182 (10.9%) patients had a positive margin. Nine patients with genotype 16 had positive margin, while in the hr HPV group, only 6 patients had positive margin.

From the Table 3 we can see how all the patients (9 cases) with positive margin and 16 HPV positive have developed a recurrence (100%), while the hr HPV patients (6 cases) only relapsed in one case. Furthermore, the HPV 16 positive women with a negative margin, developed 8 recurrences (40%). Women with hr HPV but whose margin was negative did not developed any recurrence. Table 4 show the OR.

**Table 3:** Follow-up according to margin status, HPV status and recurrence

		%	No recurrence	%	Recurrence	%
Positive Margin						
16 HPV	9	45	0	0	9	100
Hr HPV	6	30	5	83.3	1	16,6
negative HPV	5	25	5	100	0	0
Negative Margin						
16 HPV	20	12.3	12	60	8	40
Hr HPV	3	1,8	3	100	0	0
negative HPV	139	85.8	139	100	0	0

**Table 4:** Odd Ratio: recurrence after treatment

	Odds ratio (95% CL)	*P-value*
HPV 16 positive margin	45 (2.29–885.65)	.0034
HPV 16 infection	11.33 (1.25–102.93)	.0126

## Discussion

The women with CIN2+ with residual or recurring lesions are at 5 times greater risk of cancer with respect to the general population [4]. Various studies have reported that persistent HPV infection after LEEP is a risk factor for residual/recurrent disease [5].

On the other hand, the viral clearance at follow-up after conization is significatively associated with efficacy of the surgical treatment, as confirmed by Cricca et al. [6]. Authors [7], in particular, reported that the rate of persistence of HPV infection after conization for CIN 3 was approximately 20, and 46% of these patients with persistent HPV infection developed CIN relapse at 4–10 months after treatment. In our study, the rate of persistent infection from HPV 16, after LEEP, was 15.9% (29/182) and occurring within 18 months of FU.

From this study it was found that the persistence of genotype 16 is associated with a greater rate (17/18) (94.4%) (*p* < .05) of relapse post-conization of CIN 2+ lesions, respect to other genotypes with OR = 11.33 (CL95% = 1.25–102.93).

Our study further supports those studies [5] that demonstrate that the risk for residual disease or relapse is not to be overlooked, also when the margins are negative, but persistent HPV infection is present. In our case study, 40% of relapses were in women with negative margin, but with persistent HPV 16 infection.

Even more so, the margins involved in HPV 16 positive subjects is another prediction factor for relapse OR = 45 (CL 95% = 2.29–885.65).

Viral clearance at follow-up can also occur in cases of positive margins [6], only these cases do not need further treatment; 6 cases in our study became negative within 24 months of FU.

Moreover, in our study, no cases of relapse were found in women who were negative at the HPV test at 6 months, independently from the type of involvement of the margin, indicating a negative predictive value of 100% for relapse during follow-up [8].

Our results show the importance of genotyping [9] and that persistent HPV 16 infection should be considered a risk factor for the development of residual/recurrent CIN 2/3. Moreover, Authors [10] reported that HPV 16 positivity, 6 months after LEEP, was associated with a 37% increase of the absolute risk at 2 years for CIN 2+, twice that associated with HPV 18 (18.5%), and three times that of other types of oncogenes (10.8%). This result indicates that the HPV genotype should be considered in the policy of post-treatment monitoring, as suggested by many authors [11].

Furthermore, some researchers believe that when the DNA of post-treatment HPV is absent for 3 to 6 months after conization, especially in patients with a negative margin of the cone, the patients can go back to general-population screening [12].

Furthermore, the possibility of re-infection after treatment should be considered. Infact, Jung Mi Byun et al. [13] during follow-up, 70.7% of HPV infections were new and 29.3% were persistent, indicating a need to prevent reinfection after treatment and to regular follow-up for persistent HPV infection. HPV vaccination for HPV 16 type may be useful in preventing recurrence of CIN 2/3 and CIS. The efficacy of vaccination after treatment of CIN should now be investigated.

## Conclusions

The prevalence of the diverse genotypes of HPV seen in our cohort is similar to other Italian and European populations [14]. HPV 16 has been confirmed as the prevalent genotype in CIN2+ and is generally, by itself, responsible for the pathology (single genotype) [15].

In the absence of viral infection, the risk of relapse is minimal. On the other hand, the cone with negative margins in the presence of persistent HPV 16 infection has a high incidence of relapse and thus persistent infection with HPV 16 should be considered a risk factor for the development of CIN2+ relapse.

HPV vaccination for HPV 16 type may be useful in preventing recurrence of CIN 2/3 and CIS.

There are some limitations in our study that include the small sample size, the retrospective design and the limited long-term follow-up. However, follow-up is still underway.

## Data Availability

The datasets used and/or analyzed during the current study are available from the corresponding author on request.

## Abbreviations

CIN2+: All the cases of CIN3, SCC lesion
CIS: Carcinoma In Situ
FU: Follow-Up
HPV: Human Papillomavirus
hr HPV: High risk HPV
SCC: Squamous Cervical Carcinoma
